# Contradictory Role of Gadd45β in Liver Diseases

**DOI:** 10.1111/jcmm.70267

**Published:** 2024-12-09

**Authors:** Chi Wu, Xiaozhen Song, Miaoxin Zhang, Longjun Yang, Panpan Lu, Qiang Ding, Mei Liu

**Affiliations:** ^1^ Department of Gastroenterology, Tongji Hospital, Tongji Medical College Huazhong University of Science and Technology Wuhan Hubei China

**Keywords:** fatty liver, Gadd45β, hepatocellular carcinoma, hepatocellular damage, liver fibrosis

## Abstract

There are three homologous proteins (α, β and γ) in the growth arrest and DNA damage 45 (Gadd45) family. These proteins act as cellular responders to physiological and environmental stimuli. Gadd45β plays a significant role in the pathogenesis of liver diseases. Liver injury and growth stimulation increase expression of Gadd45β, which promotes cell survival, growth and proliferation in normal liver cells. By contrast, Gadd45β plays a role in promoting apoptosis and inhibiting tumour function in hepatocellular carcinoma (HCC). Currently, it is believed that Gadd45β benefits the liver through two different pathways: binding to MAPK kinase 6 (MKK6) to increase PCD induced by p38 (inhibiting tumours) or binding to constitutive androstane receptor (CAR) to jointly activate transcription of liver synthesis metabolism (promoting liver regeneration). This article aims to systematically review the role of Gadd45β in liver diseases, including its regulatory mechanism on expression and involvement in liver cell damage, inflammation, fibrosis and HCC. In conclusion, we explore the potential of targeting Gadd45β as a therapeutic strategy for liver diseases.

**Abbreviations**
AAV: adeno‐associated virus
AE: alveolar echinococcosis
AHPN: 6‐[3‐(1‐adamantyl)‐4‐hydroxyphenyl]‐2‐naphthalene carboxylic acid
AHR: aryl hydrocarbon receptor
Akt/PKB: protein kinase B
ALD: alcoholic hepatitis
ALT: alanine aminotransferase
APAP: acetaminophen
ATRA: all‐trans retinoic acid
bHLH/PAS: basic helix–loop–helix
cAMP: cyclic adenosine monophosphate
CAR: constitutive androstane receptor; Nr1i3
CCl4: tetrachloromethane
DDT: dichlorodiphenyltrichloroethane
DEN: N‐diethyinitrosamine
DZNep: 3‐deoxyadenosine A
E2F‐1: E2F transcription factor 1
EZH2: enhancer of zeste homologue 2
FF: fenofibrate
FOXO1: forkhead box proteins O1
Gadd45: growth arrest and DNA damage inducible 45 proteins
H3K27me3: histone h3 lysine 27 methylation
HCC: hepatocellular carcinoma
HCV: hepatitis C virus
HSCs: hepatic stellate cells
HSP72: heat shock protein 72
IHC: immunohistochemistry
IL1: Interleukin 1
IL6: Interleukin 6
IncRNA: long noncoding RNA
IP: immunoprecipitation
IR: insulin resistance
JNK: c‐Jun N‐terminal kinase
LPS: lipopolysaccharide
MKK4/JNKK: MAPK kinase 4/JNK kinase 1
MKK6: MAPK kinase 6
MKK7/JNKK2: MAPK kinase 7/JNK kinase 2
MOLE:
*Moringa oleifera* Lam leaf extract
MTK1/MEKK4: mitogen‐activated protein kinase kinase kinase 4
NAFLD: nonalcoholic fatty liver disease
NFκB: nuclear factor kappa B
P38: p38 MAPK
PB: phenobarbital
PCD: programmed cell death
PCN: pregnenolone‐16α‐carbonitrile
PEITC: phenethyl isothiocyanate
PGC‐1α: peroxisome proliferator‐activated receptor γ coactivator 1α
PH: partial hepatectomy
PPARα: peroxisome proliferator activated receptor alpha
PXR: pregnane X receptor; Nr1i2
qRT‐PCR: quantitative real‐time reverse‐transcription polymerase chain reaction
SAMe: S‐adenosylmethionine
SMAD: drosophila mothers against decapentaplegic protein
STAT3: signal transducer and activator of transcription 3
TAM: tumour‐associated macrophage
TCDD: 2,3,7,8‐tetrachlorodibenzo‐p‐dioxin (TCDD); dioxin
TCPOBOP: 1,4‐Bis[2‐(3,5‐dichloropyridyloxy)]benzene
TET1: ten‐eleven translocation 1
TGFβ: transforming growth factor β
TNFα: tumour necrosis factor α
TNFR1, 2: tumour necrosis factor α receptor 1, 2
WB: Western blotting

## 1 Introduction

The Gadd45 family comprises three homologous proteins (α, β and γ), known for inducing growth arrest and DNA repair. These proteins are associated with cycle arrest, apoptosis, DNA demethylation and repair, survival and response to physiological and carcinogenic stress [1]. Their function is presented by protein–protein interactions, regulating cell cycle and cellular response to stress. The distinct biological effects of each Gadd45 protein likely stem from varying induction signals and cell‐specific expression patterns. This article aims to elucidate the mechanism by which Gadd45β operates in liver diseases and assess its potential as a therapeutic target.

Gadd45β is ubiquitously expressed in murine tissues, including the liver [2]. Gadd45β protein plays a crucial role in responding to physiological and environmental stressors. Liver injury and growth stimulation induce increased expression of Gadd45β, contributing to liver homeostasis. The liver performs a diverse range of functions, including essential processes such as bile metabolism, lipid synthesis, carbohydrate metabolism and serum protein synthesis. Each of these processes requires precise regulation based on metabolic resources and specific stimuli, and is related to Gadd45β, which affects liver injury, inflammation, cell growth and proliferation [3].

## 2 Gadd45β Expression in Liver Diseases

### 2.1 Hepatocellular Damage

In various liver injuries and under the influence of hepatotoxic drugs, liver Gadd45β expression is commonly upregulated. For instance, in cases of alveolar echinococcosis (AE), infected hepatocytes exhibit continually increased Gadd45β expression [4]. Similarly, transcriptome analysis of mouse liver subjected to ischaemia–reperfusion injury revealed significantly elevated Gadd45β levels [5].

In addition to liver disease‐induced damage, the induction of Gadd45β by drug‐induced liver damage is well‐documented. Dichlorodiphenyltrichloroethane (DDT) is a persistent estrogenic organochlorine pesticide that has a promoting effect on liver tumours in rodents. Its induced liver damage is accompanied with increased Gadd45β expression [6]. The effects of methanolic coal dust extract on human HCC cell line HepG2 upregulated Gadd45β expression by up to 4.7 times [7]. Nonylphenol causes liver injury in rats, which can increase Gadd45β expression and may induce oxidative stress, thus causing toxic effects on the liver [8]. Bisphenol A treatment in rats can increase Gadd45β expression in the liver dose dependently [9]. Similarly, its substitute for manufacturing, bisphenol S, can increase Gadd45β expression in the liver, indicating that chronic exposure to bisphenol S can affect liver function in mice by inducing oxidative damage. Thus, bisphenol S may not be a safe substitute for bisphenol A [10]. The extract of *Moringa oleifera* Lam leaves (MOLE) and hydrogel nanoparticles made of starch‐MOLE‐bovine serum albumin (BSA) have a protective activity against hepatotoxicity induced by bisphenol A in male rats, which was also accompanied with Gadd45β regulation [11]. A previous study has proposed that using high‐throughput Gadd45β reporter gene testing to evaluate potential liver toxicity assesses drug‐induced DNA damage [12].

Moreover, Gadd45β could be used to evaluate liver toxicity and protective effects of drugs. Under restricted feeding (RF) conditions, all‐trans retinoic acid (ATRA) can reduce the average levels of liver ALT and Gadd45β [13]. A yeast‐derived selenium (YS) supplementation diet (1 mg/kg diet) for 100 days significantly reduced the level of Gadd45β protein in mice liver [14]. Cactus extract, mainly composed of betaine, reduces the expression of stress‐related gene Gadd45β increased by acetaminophen (APAP) [15].

In addition, autopsy and tissue analysis of offsprings of pregnant mice with ammonium perfluoro (2‐methyl‐3‐oxahexanoate) (GenX) by gavage demonstrated its induced developmental hepatotoxicity, which was also accompanied with increased Gadd45β expression in the liver [16].

Overall, Gadd45β increases during liver injury and decreases during liver recovery, used to evaluate hepatocellular damage.

### 2.2 Liver Steatosis

It is generally believed that Gadd45β plays a crucial role in lipid metabolism, but changes of its expression in liver steatosis are still uncertain. Liver Gadd45β expression was upregulated in several mouse models of obesity and diabetes [17]. In the study of the pathogenic role of dysfunctional subcutaneous white adipose tissue in the progression of nonalcoholic fatty liver disease (NAFLD), RNA sequencing analysis showed that Gadd45β expression paralleled the degree of steatosis [18]. In a study on the contribution of TNFα to insulin resistance (IR), the authors compared the transcriptional profiles of rat H‐411E hepatocytes exposed to insulin with or without TNFα, in which Gadd45β expression was upregulated by insulin and then reversed by TNFα [19].

By contrast, bioinformatics analysis has shown that the relative expression level of Gadd45β in fatty liver model cells is lower than that in the control group [20]. An analysis of the GEO dataset has revealed that Gadd45β expression was significantly reduced in the liver of patients with NAFLD and that the mRNA of Gadd45β was significantly reduced in the liver of mice fed with a high‐fat and high‐fructose diet (HFHF) [21]. Gadd45β expression is significantly reduced in NAFLD‐related HCC [22], which may be related to its mediated anti‐cancer effect.

The different manifestations of Gadd45β in fatty liver suggest a complex mechanism of Gadd45β regulating lipid metabolism. This opposite change of expression may be related to the various pathways of Gadd45β. Furthermore, as fatty liver progresses to hepatitis and liver fibrosis, the functional focus of Gadd45β changes with time.

### 2.3 Liver Fibrosis

Significant downregulation of the expression of Gadd45α, rather than of Gadd45β or Gadd45γ, was detected in mouse liver fibrosis tissue induced by CCl4 and isolated HSCs, accompanied with activation of the TGFβ–SMAD signalling pathway [23]. Currently, the expression of Gadd45β in liver fibrosis is unclear.

### 2.4 Hepatocellular Carcinoma

Unlike the variable expressions of Gadd45β in steatosis, the gene expression changes in HCC are very consistent. Gadd45β expression in HCC is reduced [24] and gradually decreases with the progression of liver cancer [25]. A previous analysis has revealed that Gadd45β expression is induced or inhibited in a unique pattern of specific carcinogens, without being altered by non‐carcinogens [26]. Although the liver cancer inducers diethylnitrosamine (DEN) and ethylnitrosourea (ENU) significantly and dose‐dependently increase Gadd45β expression 4 h after treatment of mouse liver [27], this should be related to the early stress response caused by hepatocellular injury and does not affect the expression of advanced tumours after injury. The noncoding RNA associated with Gadd45β also showed corresponding expression changes, with miR‐423‐5p showing a high expression in HCC and IncRNA FENDRR and Gadd45β exhibiting a low expression in HCC [28]. RNA sequencing analysis of 370 samples with patient information from the Cancer Genome Atlas (TCGA) dataset indicated that most genes affecting prognosis were highly expressed in HCC cell lines, whereas Gadd45β expression was downregulated [29].

Owing to the strong correlation between Gadd45β and HCC, more data utilising Gadd45β as an evaluation indicator are presented. Chemical sensitive genes, including Gadd45β that may become therapeutic targets for HCC were identified in patients with HCC analysed with a connectivity map (CMap) analysis report [30].

Phenethyl isothiocyanate (PEITC) is a component of cruciferous vegetables with proven anti‐cancer activity in many cancer models, and its benefit has also been associated with increased Gadd45β expression [31].

In various models of immunotherapy‐resistant solid cancers, including HCC and ovarian adenocarcinoma, Gadd45β loss in bone marrow cells restores the activation of pro‐inflammatory tumour‐associated macrophages (TAMs) and tumour immune infiltration thereby reducing tumour occurrence, showing a direct relationship between elevated expression of Gadd45β and poor prognosis [32]. Despite exhibiting contradictory results in tumour cells and immune cells, the impact of Gadd45β on tumour cells comes not only from the tumour cells themselves, but also through the assistance of other immune cells.

Overall, Gadd45β decreases in HCC and may serve as a target for cancer treatment (Table 1).

**TABLE 1:** Expressions of Gadd45β in liver diseases.

Liver disease	Gadd45β analysis method	Sample	Gadd45β expression	Findings	Reference
Hepatocellular damage	WB, qRT‐PCR, IHC	Mouse (liver tissue)	Increase	Gadd45β increased in infiltrating lymphocytes, fibroblast‐like cells and hepatocytes with infection of alveolar echinococcosis.	Zhang et al. [4]
	RNA‐Seq, qRT‐PCR	Mouse (liver tissue)	Increase	Gadd45β increased after hepatic ischaemia/reperfusion injury.	Zhang et al. [5]
	Microarray, qRT‐PCR	Mouse (liver tissue)	Increase	o, p'‐DDT‐induced hepatic Gadd45β in mice.	Kiyosawa et al. [6]
	qRT‐PCR	Human (HepG2 cell)	Increase	Gadd45β expression increased in HepG2 with a methanolic coal dust extract.	Guerrero‐Castilla et al. [7]
	qRT‐PCR	Rat (liver tissue)	Increase	Bisphenol A increased Gadd45β expression.	Kazemi et al. [8]
	qRT‐PCR	Rat (liver tissue)	Increase	Nonylphenol can increase Gadd45β through induction of oxidative stress.	Kazemi et al. [9]
	qRT‐PCR	Mouse (liver tissue)	Increase	Bisphenol S induced oxidative stress in the liver of mice by increasing Gadd45β expression.	Zhang et al. [10]
	qRT‐PCR	Rat (liver tissue)	Decrease	Hepatoprotective activity of MOLE against bisphenol A‐induced liver toxicity on maintaining homeostasis of Gadd45β.	Abou El‐Naga et al. [11]
	qRT‐PCR	Mouse (jejunum and liver tissue)	Increase	Under RF, ATRA reduced jejunum Gadd45β but increased liver Gadd45β mRNA.	Sherman et al. [13]
	Microarray, qRT‐PCR, WB	Mouse (liver tissue)	Decrease	Yeast‐derived selenium significantly reduced Gadd45β expression in the liver.	Barger et al. [14]
	qRT‐PCR	Rat (isolated hepatocyte and liver tissue)	Decrease	Opuntia extracts reduced Gadd45β expression induced by acetaminophen.	González‐Ponce et al. [15]
	RNA sequencing, WB	Mouse (liver tissue)	Increase	Gadd45β was significantly increased by GenX‐induced developmental hepatotoxicity.	Zhang et al. [16]
Liver steatosis	RNA‐Seq, qRT‐PCR, WB	Mouse (liver tissue)	Increase	Gadd45β expression increased in mice liver under fasting conditions. Gadd45β expression was increased in MPHs following to dexamethasone exposure.	Wu et al. [17]
	RNA‐sequencing	Human (MSC‐derived adipocytes)	Increase	Gadd45β expression paralleled steatosis degree.	Lopez‐Yus et al. [18]
	RNA‐sequencing	Rat (H‐411E liver cell)	Increase	Gadd45β expression increased with insulin and then reversed by TNFα.	Solomon et al. [19]
	qRT‐PCR, microarray	Human (liver tissue and QSG‐7011 cell)	Decrease	Gadd45β expression was lower in QSG‐7011 fatty liver model cells than in the control.	Liao et al. [20]
	GEO dataset, qRT‐PCR	Human (liver tissue), mouse (liver tissue)	Decrease	Gadd45β expression was decreased in the livers of patients with NAFLD and in the mouse model.	Dong et al. [21]
	GEO dataset, qRT‐PCR	Human (liver tissue), mouse (liver tissue)	Decrease	Gadd45β was significantly decreased in NAFLD‐associated HCC.	Zhang et al. [22]
Hepatocellular carcinoma	Microarray, Northern blot, qRT‐PCR, IHC	Human (liver tissue, HepG2 and Hep3B cells)	Decrease	Gadd45β staining in HCC was significantly decreased.	Qiu et al. [24]
	qRT‐PCR	Human (liver tissue)	Decrease	Gadd45β gradually decreased with HCC development.	Yoo et al. [25]
	Microarray, qRT‐PCR	Mouse (liver tissue)	Increase	Gadd45β was induced in unique patterns for specific carcinogens and not altered by the non‐carcinogens.	Iida et al. [26]
	qRT‐PCR	Mouse (liver tissue)	Increase	Gadd45β dose dependently increased at 4 h for both carcinogens.	Watanabe et al. [27]
	qRT‐PCR, WB	Human (liver tissue)	Decrease	Gadd45β expression decreased in HCC.	Yu et al. [28]
	RNA‐sequencing, qRT‐PCR	Human (liver tissue, Huh‐7, HepG2 and LO2 cells)	Decrease	Gadd45β expression was decreased in HCC cell lines.	Shi et al. [29]
	Microarray	Human (liver tissue)	Decrease	Gadd45β expression was downregulated in HCC patient samples.	Yang et al. [30]
	Microarray	Rat (liver tissue)	Increase	Gadd45β expression increased with PEITC.	Telang et al. [31]

## 3 Mechanism of Gadd45β in Liver Diseases

### 3.1 Hepatocellular Damage

The mechanism of action of Gadd45β in liver injury is intricate and variable. Gadd45β^−/−^ mice exhibit significantly decreased proliferation during liver regeneration, underscoring the vital role of this protein's adaptive response to liver mass loss [33]. In fact, Gadd45β^−/−^ has different effects on the proliferation induced by two CAR activators (TCPOBOP and PB). Although Gadd45β loss increased the proliferation induced by TCPOBOP [34], it decreased the proliferation induced by phenobarbital (PB) [35]. The difference observed between these two effects results from the indirect activation of CAR by PB through the p38 MAPK signal, which is significantly reduced without Gadd45β. By contrast, Gadd45β affects CAR activation and nuclear translocation mediated by PB rather than by TCPOBOP.

After partial hepatectomy (PH), Gadd45β^−/−^ can induce liver damage leading to substantial mortality due to unbridled JNK activation [33]. Gadd45β deficiency in knockout mice cannot be compensated by increasing the level of Gadd45α or Gadd45γ, which confirms their independent transcriptional regulation and indicates that the effect of PH completely depends on Gadd45β deficiency. By binding and inhibiting Jun kinase MKK7/JNKK2, Gadd45β inhibits JNK activation, thereby improving the potential damage mediated by TNFα signalling [36, 37, 38]. This is a crucial pathway, as TNFα initiates liver regeneration, whereas Tnfr1^−/−^ mice cannot undergo liver regeneration after PH [39].

Gadd45β loss does not eliminate regeneration completely, but 56% of mice die after PH because of severe cell damage and inflammation [33]. Knocking out JNK2 and introducing it into the Gadd45β^−/−^ mice completely restored their liver regeneration [33]. The experiment also confirmed the anti‐proliferative effect of JNK2 in extracted hepatocytes [40]. JNK1 activates cell proliferation by phosphorylating Jun, whereas JNK2 actually reduces the cellular level of Jun and its activation of cell proliferation [41]. Therefore, the key function of Gadd45β in stimulating liver regeneration is to regulate the destructive effect of TNFα signalling, as inhibiting JNK2 activation shifts the balance towards protective growth stimulation response.

### 3.2 Liver Steatosis

The different manifestations of Gadd45β in fatty liver reveal its complex regulatory mechanisms in lipid metabolism. Gadd45β overexpression in vivo or in cultured hepatocytes activates hepatocyte generation and increases hepatic glucose production (HGP). Conversely, liver‐specific Gadd45β gene knockout mice exhibit hyperglycaemia resistance induced by high‐fat diet or steroids [17], which is attributed to the joint promotion of DNA demethylation of PGC‐1α promoter by Gadd45β and TET1, with stimulating PGC‐1α expression and promoting tumour development or hyperglycaemia. Gadd45β does not promote Akt‐mediated phosphorylation of forkhead box protein O1 (FoxO1) and forskolin‐induced cAMP response element‐binding protein (CREB); instead, it heightens FoxO1's transcriptional activity by enhancing its protein stability, acting as an essential regulator of hepatic gluconeogenesis [42].

It is worthy mentioned that Gadd45β can help alleviate fatty liver. Gadd45β is stabilised by interacting with HSP72 and can reduce pathological changes related to NAFLD [21]. Similarly, weight gain reduction and improved IR triggered by the CAR agonist TCPOBOP were significantly hindered in Gadd45β^−/−^ mice. The inhibition of liver adipogenesis and inflammation caused by TCPOBOP observed in wild‐type mice was largely eliminated in Gadd45β^−/−^ mice, with CAR activation mitigating hyperglycaemia by hindering glucose production and bolstering hepatic glucose uptake [43]. The decrease of Gadd45β expression and increase of lipid uptake were also observed in mice with obesity or diabetes. In both scenarios, Gadd45β overexpression reversed these alterations. The relationship between CAR and Gadd45β in lipid metabolism or diabetes has been confirmed by a study on high‐fat diet mice, in which CAR activation has the effect of anti‐diabetes, reducing fat and improving IR [44]. In this model, the absence of Gadd45β nearly entirely nullified CAR's metabolic benefits [43]. The impact of Gadd45β on metabolism gradually changes during the progression of fatty liver, and its impact on cell damage and inflammation dominates the subsequent progression of fatty liver inflammation. Thus, the regulatory network complicates the assessment of Gadd45β's benefits in fatty liver disease.

### 3.3 Inflammation

With Gadd45β increasing, lipopolysaccharide (LPS) treatment activates the NFκB signalling pathway in the liver, which induces DNA damage. Several inhibitors of the NFκB signalling pathway, including dexamethasone, thalidomide and proteasome inhibitor bortezomib, can reduce LPS‐induced Gadd45β expression [45]. In the liver of bortezomib‐treated mice, a reverse correlation between luciferase activity and Gadd45β mRNA level was observed, which may be caused by severe liver toxicity due to the combination therapy of bortezomib and LPS. Gadd45β is generally considered a protein that responds to and alleviates inflammation.

Previous studies have analysed Gadd45β for assessing hepatitis status. An experiment has revealed that with ethanol, quercetin, epigallocatechin gallate (EGCG), catechin and betaine downregulated Gadd45β expression, which also explains the DNA demethylation of upregulated genes in the ALD model [46]. To date, a definite regulatory effect of Gadd45β on hepatitis has not been determined. Gadd45β is only an accompanying genetic changing during the occurrence of hepatitis, serving as a stress response molecule to alleviate cellular damage caused by inflammation.

### 3.4 Liver Fibrosis

Liver fibrosis is a crucial process in the progression of liver diseases, with Gadd45β being involved in such process. The current research has not fully elucidated the exact function and mechanism of Gadd45β in liver fibrosis, and future research may further reveal its potential value in the treatment of liver diseases.

Recent studies have identified that Gadd45β deficiency promotes senescence and alleviates liver fibrosis, whereas Gadd45α can play a protective role in liver fibrosis. Compared with the wild‐type mice, Gadd45β^−/−^ mice have less cellular structural damage (fibrotic scars) and a higher tendency for embryonic fibroblasts (MEFs) to senescence, thereby alleviating fibrosis [47]. The therapeutic effect of 3‐deoxyadenosine A (DZNep) as an epigenetic drug in liver fibrosis is related to Gadd45β regulation by EZH2 [48]. Inhibition of EZH2 helps to alleviate liver fibrosis by reducing H3K27me3 recruitment and increasing the expression of cell cycle regulators, including Gadd45β. This mainly focuses on the regulatory effect of Gadd45β on the cell cycle, which is considered as a cell‐repairing factor for reducing the occurrence of fibrosis here. This is inconsistent with the results in genetic animals, which suggests the involvement of Gadd45β in the occurrence of liver fibrosis and its incongruous role beyond regulating the cell cycle.

After considering various articles exploring expression levels and mechanisms of Gadd45β, most studies currently indicate that Gadd45β can promote liver fibrosis, although its molecular regulatory mechanism remains unknown.

### 3.5 Hepatocellular Carcinoma

HCC is a severe liver disease and Gadd45β also plays an important role in its occurrence and development. The promoter of Gadd45β is highly methylated in numerous HCC cases [24, 49, 50, 51], which is a change associated with low or absent expression. Pro‐apoptotic and anticancer effects of Gadd45β are weakened in HCC.

By contrast, the involvement of Gadd45β in important stages of HCC progression has also been reported. PB is a non‐genotoxic carcinogen that can induce liver proliferation in rodents and promote the occurrence of HCC. During the long‐term toxicity process of PB, Gadd45β showed inconsistency. Early drug damage led to increased Gadd45β expression, whereas a decrease in Gadd45β was witnessed in subsequent tumour development. Compared with wild‐type mice, Gadd45β^−/−^ mice exhibited less liver proliferation and tumour development and significantly reduced hepatocyte proliferation induced by PB. PB‐induced proliferation persisted in C57BL/6 male mice until 48 h after PB injection, whereas this did not persist in Gadd45β^−/−^ male and female mice and C57BL/6 female mice. These data reveal the interaction between nuclear receptor CAR and Gadd45β to inhibit p38MAPK signalling and induce hepatocyte proliferation in male mice [35]. The study used Gadd45β^−/−^ mice treated with DEN at 5 weeks of age and fed with drinking water supplemented with PB from 7 to 57 weeks of age. Compared with wild‐type mice, Gadd45β^−/−^ mice in the PB‐treated group did not develop HCC. The increase in liver weight in wild‐type mice was more significant than that in Gadd45β^−/−^ mice, thus suggesting the indispensable role of Gadd45β in HCC development by regulating irisin/Fndc5 and Tgfbr2 [52]. Tsc‐22 is a tumour suppressor gene that represents a novel kind of transcription factor that has transcriptional repressor activity and may suppress Gadd45β which may contribute to an early antiapoptotic response [53]. Unfortunately, no drugs have used this as a target to inhibit tumour growth or prevent tumour progression by inhibiting the function of Gadd45β.

The study confirmed the lower expression of Gadd45β in human HCC tissue and cell lines compared to adjacent liver tissue and normal hepatocytes. Moreover, Gadd45β can inhibit the stemness of HCC cells and enhance apoptosis induced by chemotherapy for cancer. 5‐Azocytidine can reduce the stemness of SMMC‐7721 and Hep‐3B cells and enhance their sensitivity to cisplatin [51]. Therefore, Gadd45β may become a potential target for enhancing chemotherapy sensitivity in HCC.

Here are some attempts to influence Gadd45β as a treatment strategy. The novel retinoic acid AHPN has been demonstrated to inhibit cell growth and induce apoptosis in several human HCC cell lines. After AHPN treatment, Gadd45β expression rapidly increased in many cancer cell lines [54]. S‐adenosylmethionine (SAMe) induces liver protection mediated by Gadd45β through NFκB, which is associated with p53 and used to correct the downregulation of Gadd45β in HCC cell lines [55]. Both oxaliplatin and sorafenib can induce Gadd45β production in HepG2 and Hep3B cells dose‐dependently, with rapid and direct cytotoxic effects [56]. Moreover, both oxaliplatin and sorafenib can boost the transcriptional activity of NFκB and E2F‐1. Consequently, SAMe selectively triggers Gadd45β induction via the NFκB pathway in HepG2 cells, resulting in gradual and indirect cytotoxic effects. This induction of Gadd45β by SAMe not only inhibits the proliferation of HCC cells but also safeguards normal hepatocytes from apoptosis while promoting their regeneration [57]. Fisetin is a naturally occurring flavonoid that induces growth inhibition and apoptosis in HCC (HepG2) cell lines. Its anti‐tumour effects primarily work through the modulation of various signalling pathways and activation of Gadd45β [58]. Meanwhile, ZY0511 has emerged as a promising therapeutic agent for HCC by upregulating Gadd45β, thereby proposing a novel combination strategy for treating liver diseases [59]. The role of Gadd45β in facilitating sorafenib‐triggered apoptosis in HCC cells requires further research to validate its potential in predicting the efficacy of sorafenib [60]. Moreover, a combined treatment regimen of depsipeptide and 5‐azacytidine, in sequence, has demonstrated a synergistic effect on reactivating Gadd45β in HepG2 cells [61]. LncRNA FENDRR upregulates Gadd45β by competitively binding to miR‐423‐5p and inhibits immune escape mediated by Treg in HCC cells [28]. This evidence suggests targeting Gadd45β as a potential strategy for treating HCC, by enhancing tumour apoptosis through Gadd45β upregulation.

The current strategies favour upregulating Gadd45β to enhance tumour apoptosis rather than inhibiting its function to suppress tumour growth in HCC treatment. This reflects the general preference for Gadd45β, which is widely recognised for promoting tumour apoptosis and regulating cell cycle. Nevertheless, the exact influence of Gadd45β on tumour initiation remains unclear. Thus, we can simply assume that Gadd45β has the function of promoting cell apoptosis and therefore can inhibit tumours, whereas the subsequent effect of binding with CAR promotes liver regeneration and tumour development. However, this cannot explain why Gadd45β can also inhibit apoptosis through the MKK7 pathway without CAR action. From the perspective of reducing oncogenesis, inhibiting Gadd45β as a method for preventing HCC oncogenesis has limited prospects and acceptance compared with post‐tumour intervention treatment (Table 2).

**TABLE 2:** Mechanisms of Gadd45β in liver diseases.

Liver disease	Gadd45β analysis method	Gadd45β function	Findings	Reference
Hepatocellular damage	WB, IP	Pro‐apoptosis	CAR interacts with Gadd45β to repress p38 MAPK signalling and decrease hepatocyte proliferation in male mice.	Hori et al. [35]
	WB	Anti‐apoptosis	Upregulation of Gadd45β/myd118 by NF‐kB downregulates JNK signalling induced by the TNF receptor (TNF‐R).	Smaele et al. [36] Bubici et al. [75]
	qRT‐PCR, WB	Pro‐proliferation	Gadd45β^−/−^ mice keep proliferation following TCPOBOP, but growth delays. Early transcriptional stimulation of CAR target genes was weaker in Gadd45β^−/−^ mice.	Tian et al. [34]
	—	Anti‐apoptosis	Gadd45β^−/−^ mice exhibited decreased hepatocyte proliferation and increased PCD. JNK activity was increased and sustained in livers of Gadd45β^−/−^ mice.	apa et al. [33]
Liver steatosis	RNA‐Seq, qRT‐PCR, WB	Pro‐gluconeogenesis	Gadd45β promotes DNA demethylation of PGC‐1α promoter in binding with TET1, thereby stimulating PGC‐1α expression to promote gluconeogenesis and hyperglycaemia.	Wu et al. [17]
	qRT‐PCR, WB, IP	Pro‐gluconeogenesis	Gadd45β increased FoxO1 transcriptional activity by enhanced protein stability of FoxO1.	Kim et al. [42]
	qRT‐PCR, WB, IP	Anti‐steatosis	HSP72 interacted with Gadd45β to prevent it from being degraded by the proteasome, contributing to regulating TG synthesis and insulin signalling pathway.	Dong et al. [21]
	—	Anti‐lipogenesis, anti‐gluconeogenesis	Reduced body weight gain and improved insulin sensitivity by the CAR agonist TCPOBOP were decreased in Gadd45β^−/−^ mice. Inhibition of hepatic lipogenesis, gluconeogenesis and adipose inflammation disappeared in Gadd45β^−/−^ mice.	Cai et al. [43]
Inflammation	qRT‐PCR, Northern blot	Pro‐inflammation	NFκB signalling involves induction of Gadd45β expression.	Zhang et al. [45]
	qRT‐PCR	Pro‐Inflammation	All four agents decreased Gadd45β in the presence of ethanol, which could explain the mechanism of DNA demethylation of genes increasing observed in ALD.	Oliva et al. [46]
Liver fibrosis	—	Pro‐fibrosis	Fibrosis of Gadd45β^−/−^ mice is reduced, MEFs of Gadd45β^−/−^ mouse show a greater tendency to senescence, thereby alleviating fibrosis.	Zaidi et al. [47]
	qRT‐PCR, WB	Anti‐fibrosis	EZH2 inhibition decreased H3K27me3 recruitment at target Gadd45β and increased its expression.	Jiang et al. [48]
Hepatocellular carcinoma	qRT‐PCR, WB	Pro‐apoptosis	Downregulation of miR‐423‐5p increased the apoptosis of HCC cells by targeting Gadd45β.	Yu et al. [28]
	qRT‐PCR	Anti‐inflammation, pro‐oncogenesis	Gadd45β inhibition in myeloid cells restored activation of proinflammatory TAMs, thereby declining oncogenesis.	Verzella et al. [32]
	qRT‐PCR, WB	Pro‐apoptosis	Inhibition of Gadd45β suppresses the TGFβ‐induced delayed p38 activation, whereas overexpression of Gadd45β activates the p38 MAPK by MTK1.	Qiu et al. [49] Takekawa et al. [37]
	qRT‐PCR, WB	Anti‐inflammation, anti‐oncogenesis	Decreased Gadd45β expression leads to aberrant cell cycle arrest and declined DNA repair.	Higgs et al. [50]
	qRT‐PCR, WB	Pro‐apoptosis	Gadd45β inhibited the growth of HCC cells, promoting the chemotherapy‐induced apoptosis.	Hou et al. [51]
	IP	Pro‐apoptosis	CAR interacts with Gadd45β to curb p38 MAPK signalling. Gadd45β enhances MKK6‐mediated p38 MAPK phosphorylation.	Hori et al. [35]
	qRT‐PCR	Pro‐oncogenesis	Tgfbr2 and irisin/Fndc5, were increased in PB‐treated wild type mice without significant increase in Gadd45β^−/−^ mice.	Hori et al. [52]
	qRT‐PCR, microarray	Pro‐apoptosis	Tsc‐22 is a suppressor of Gadd45βwhich may contribute to an early antiapoptotic response.	Iida et al. [53]

## 4 Regulatory Mechanism of Gadd45β Expression

**FIGURE 1:**
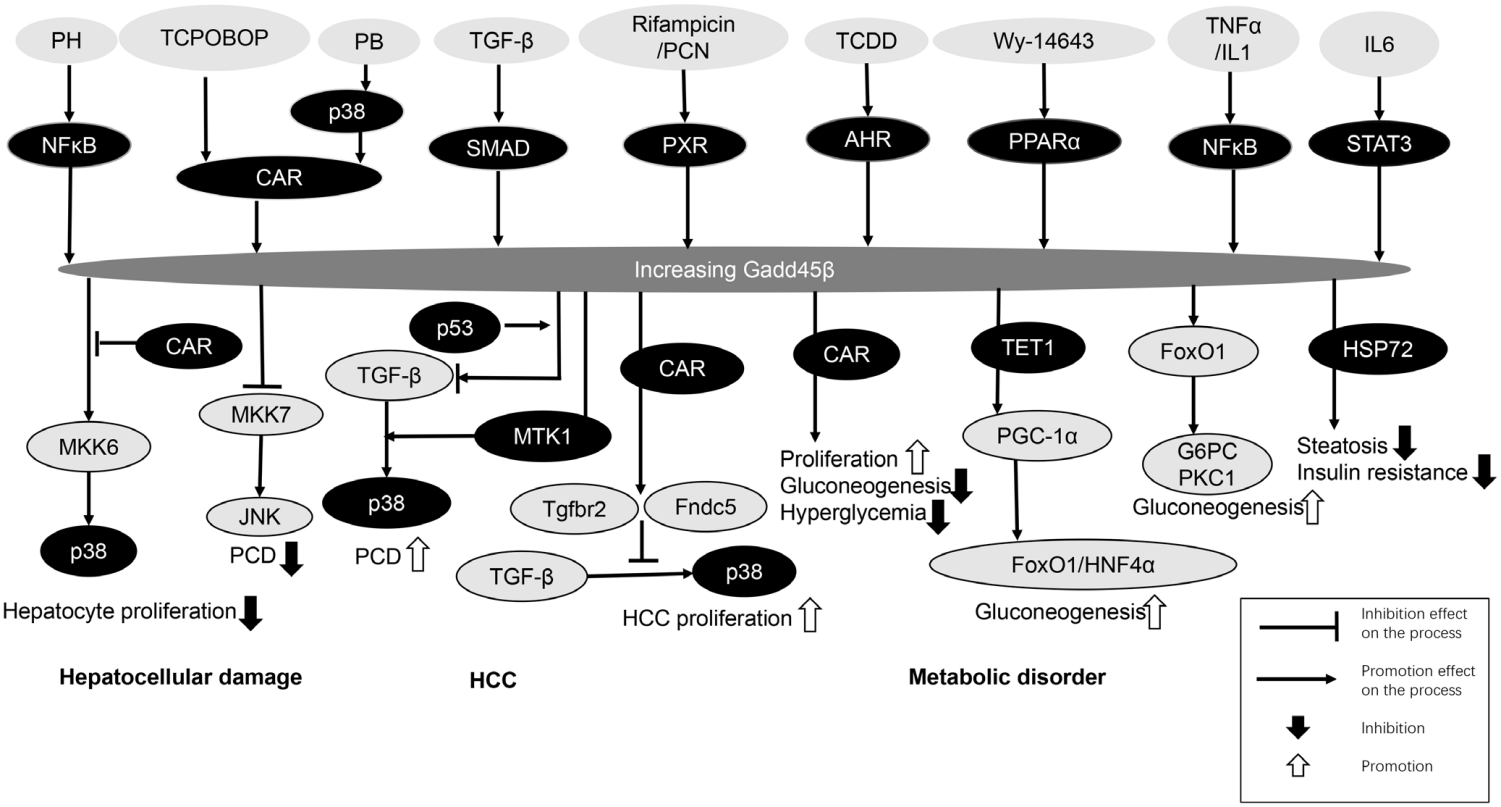
Expression regulation and mechanisms of growth arrest and DNA damage 45β (Gadd45β) in liver diseases. Several main pathways for inducing Gadd45β expression are shown. The molecular mechanisms of Gadd45β in hepatocellular damage, metabolic disorder and hepatocellular carcinoma (HCC) are shown. It is worth noting that the molecular mechanisms of Gadd45β in inflammation and fibrosis remain unclear.

Gadd45β expression is regulated by various factors, including transcription factors, epigenetic modifications and microRNA. These complex regulatory mechanisms ensure that Gadd45β is expressed at the appropriate time and location (Figure 1).

Gadd45β, as one of the most strongly induced genes in liver cell proliferation responses, is robustly induced in two primary types of responses in mice: regeneration and compensatory proliferation of liver cells after its loss, as well as hyperplasia caused by exogenous activators of drugs and constitutive androstane receptors (CARs) [62]. The induction of Gadd45β is remarkably potent, with a 70‐fold increase after PH and a 100‐fold increase after CAR stimulation with compound TCPOBOP [34, 62, 63, 64]. PB, a non‐ligand reagent with activating CAR, strongly increases Gadd45β expression [65, 66]. Regulatory effects of TGFβ–SMAD and pregnane X receptor (PXR) on Gadd45β in human liver and HCC cells have also been confirmed [37].

In addition to CAR, the following two exogenous receptors, which minimally affect hepatocyte proliferation, regulate Gadd45β: aryl hydrocarbon receptor (AHR), activated by 2,3,7,8‐tetrachlorodibenzo‐p‐dioxin (TCDD), and PXR, activated by rifampicin in humans and pregnenolone‐16α‐carbonitrile (PCN) in mice [67, 68]. TCDD stimulates Gadd45β expression and induces AHR binding to the region near the promoter [69]. Rifampicin stimulates Gadd45β expression and induces PXR binding to the region near the promoter in humans [68], whereas PXR does not induce binding near the Gadd45β promoter in mice [70]. In fact, human CAR stimulation is also uncertain as PB does not increase Gadd45β expression in mouse livers filled with human hepatocytes [71]. Moreover, peroxisome proliferator‐activated receptor alpha (PPARα) induces Gadd45β expression in mice treated with the activator Wy‐14643 [72]. Conversely, oxidative stress analysis in rat liver cells treated with PPARα agonist fenofibrate (FF) has revealed significant Gadd45β downregulation [73]. This may be related to the different activation pathways of Gadd45β in normal hepatocytes and HCC cells.

Distinct transcriptional mechanisms mediate increased Gadd45β expression during hepatocyte regeneration and proliferation. Liver damage activates signalling pathways such as TNFα–NFκB and TGFβ–SMAD, promoting Gadd45β transcription. PH rapidly activates NFκB [74], which specifically binds to the upstream region near the Gadd45β promoter and strongly activates transcription [75, 76]. This PH‐induced Gadd45β expression is compromised in Tnfr1^−/−^ mice, validating this relationship [33]. SAMe‐induced Gadd45β expression is also related to NFκB [56]. A previous study has indicated that both IL1‐NFκB and IL6‐STAT3 induced Gadd45β expression in hepatocytes, and their effects were superimposed [77]. TGFβ, an important inducer of Gadd45β transcription [78], mediates early liver regeneration and is released into local circulation within 1 h after PH [79]. TGFβ activates Smad3, which in turn activates Gadd45β transcription via enhancers [80]. Furthermore, fasting induces Gadd45β expression in the liver, correlating with alterations in lipid metabolism and implications for diabetes [81]. Fasting shifts lipid metabolism, with Gadd45β^−/−^ mice displaying significantly altered lipid uptake, underscoring the direct impact of Gadd45β on this response.

## 5 Discussion

Although Gadd45 proteins are very similar and may share almost all functions, there are some slight differences among them. Their various biological roles in the liver reflect many induction processes, with Gadd45β being one of the most prominent proteins in the liver. Gadd45β exhibits various effects in different liver models. Its positive effects are about promoting proliferation, growth, and cell survival while its negative effects include inhibiting cell proliferation and stimulating cell apoptosis (Figure 1). Each action evidently depends on the environment and molecular chaperones of interactions. Despite the functional double‐edged sword, the response of Gadd45β in hepatocytes has a unified characteristic, and the significantly elevated synthesis in the early stage reflects the importance of Gadd45β for rapid adaptation.

Gadd45β is involved in liver cell damage, inflammation, fibrosis and HCC, playing an important role in liver diseases. Although significant progress in elucidating the role of Gadd45β in liver diseases has been made, its specific mechanism of action and potential as a therapeutic target remain unclear. Future research should further explore its role in different types of liver diseases and its interactions with other signalling pathways. In addition, the effectiveness and safety of targeting Gadd45β in clinical applications need further evaluation. With further research, Gadd45β may become an important therapeutic target for liver diseases, bringing new hope to patients.

## Author Contributions

**Chi Wu:** conceptualization (lead), writing – original draft (lead), writing – review and editing (lead). **Xiaozhen Song:** writing – review and editing (equal). **Miaoxin Zhang:** writing – review and editing (equal). **Longjun Yang:** conceptualization (supporting). **Panpan Lu:** data curation (equal). **Qiang Ding:** conceptualization (supporting), funding acquisition (equal). **Mei Liu:** funding acquisition (lead).

## Ethics Statement

The authors have nothing to report.

## Consent

The authors have nothing to report.

## Conflicts of Interest

The authors declare no conflicts of interest.

## Data Availability

The authors have nothing to report.
